# Effects of Resistant Dextrin from Potato Starch on the Growth Dynamics of Selected Co-Cultured Strains of Gastrointestinal Bacteria and the Activity of Fecal Enzymes

**DOI:** 10.3390/nu14102158

**Published:** 2022-05-22

**Authors:** Michał Włodarczyk, Katarzyna Śliżewska, Renata Barczyńska, Janusz Kapuśniak

**Affiliations:** 1 Department of Biotechnology and Food Sciences, Institute of Fermentation Technology and Microbiology, Technical University of Lodz, Wolczanska 171/173, 90-924 Łódź, Poland; 2 Department of Dietetics and Food Studies, Faculty of Science and Technology, Jan Dlugosz University, Armii Krajowej 13/15, 42-200 Czestochowa, Poland; r.barczynska-felusiak@ujd.edu.pl (R.B.); j.kapusniak@ujd.edu.pl (J.K.)

**Keywords:** resistant dextrin, prebiotic, gut microbiota, bacterial enzymes

## Abstract

Preparations of resistant dextrins have become an interesting topic of research due to their properties, which bear resemblance those of prebiotics, e.g., the improvement of metabolic parameters, increased efficiency of the immune system and induction of vitamin production. The aim of this study was to investigate the effects of the resistant dextrin produced from potato starch on the growth dynamics of typical gastrointestinal microbiota and the activity of fecal enzymes in order to assess a possible exhibition of prebiotic properties. In the study, in vitro cultivation of co-cultures of *Lactobacillus*, *Bifidobacterium*, *E. coli*, *Enterococcus*, *Clostridium* and *Bacteroides* spp. was conducted on media enriched with the resistant dextrin. The CFU/mL for each strain was measured in time periods of 24, 48, 72, 96 and 168 h. Furthermore, the activities of α-glucosidase, α-galactosidase, β-glucosidase, β-galactosidase and β-glucuronidase were determined using spectrophotometric methods at a wavelength of 400 nm. The results show that the resistant dextrin can be utilized as a source of carbon for the growth of intestinal bacteria. Moreover, the results revealed that, after 168 h of cultivation, it enhances the viability of probiotic strains of *Lactobacillus* and *Bifidobacterium* spp. and decreases the growth of other intestinal strains (*Clostridium*, *Escherichia coli*, *Enterococcus* and *Bacteroides*), which is demonstrated by a high Prebiotic Index (*p* < 0.05). Furthermore, there was no significant change in the pH of the cultures; however, the pace of the pH decrease during the cultivation was slower in the case of culture with resistant dextrin. Furthermore, it was revealed that usage of the resistant dextrin as a medium additive noticeably lowered the activities of β-glucosidase and β-glucuronidase compared to the control (*p* < 0.05), whereas the activities of the other fecal enzymes were affected to a lesser degree. The resistant dextrins derived from potato starch are a suitable prebiotic candidate as they promote the growth of beneficial strains of gut bacteria and improve health markers, such as the activity of fecal enzymes. Nevertheless, additional in vivo research is necessary to further assess the suspected health-promoting properties.

## 1. Introduction

The large intestine is a complex ecosystem of microorganisms, which play important roles in processing the nutrients supplied to the body. The colon microflora is dominated by strictly anaerobic bacteria of the genera *Bacteroides*, *Clostridium*, *Fusobacterium* and *Bifidobacterium* [1]. Many species of bacteria belonging to the colon ecosystem can cause disturbances in the functioning of the digestive tract, however, only if they become the dominant microflora [2]. 

The complex of intestinal microorganisms may contribute to the formation of cancers of the kidneys, liver and gastrointestinal tract (in particular the large intestine). As colorectal cancer is the third most common cancer in Western countries and the second leading cause of death in the United States [3], it is of high importance to maintain a proper balance of the intestinal microbiota. Diet can influence the activity of the intestinal microorganisms in various ways. First, dietary substrates may induce certain microorganisms in the large intestine to increase enzymatic activity [4,5]. In addition, diet can increase the number of certain species and types of bacteria, causing changes in the composition of the gut microbial community [6].

The enzymatic activity of the large intestine microorganisms may result in the formation of products that are toxic or potentially harmful to the body [7]. The enzymes of the intestinal bacteria, which mainly belong to the classes of reductases and hydrolases, are often involved in the synthesis of carcinogens and other toxic substances. Among them, the highest activity is shown by β-glucuronidase (EC 3.2.1.31), β-glucosidase (EC 3.2.1.21), β-galactosidase (EC 3.2.1.23) and nitroreductase (EC 1.7.1.1) [8].

A comparison of the enzymatic activity of *Clostridium* bacteria isolated from the feces of patients and bacteria isolated from healthy people indicates an increased activity of azoreductase, nitroreductase, β-glucuronidase and β-glucosidase in people diagnosed with colorectal cancer [9,10].

This is confirmed by other reports and proves that, among the microorganisms of the large intestine, the highest activity of β-glucuronidase and β-glucosidase is presented by *Clostridium* spp. Moreover, it was observed that the number of bacteria of the genus *Clostridium* was significantly increased in patients with colorectal cancer [11,12].

Epidemiological studies show that both antibiotics and a diet rich in fats and proteins in the form of red meat may increase the number of *Clostridium* spp., *Bacteroides* spp., *Escherichia coli*, *Streptococci* and *Enterococci* bacteria in the large intestine [13,14]. Specific changes in the composition of intestinal microflora, where less desirable species start to dominate the environment is often related to increased activity of β-glucuronidase, β-glucosidase, nitroreductase and azoreductase [15]. For this reason, a properly composed diet is crucial as it modulates the diversity of intestinal microorganisms and thus controls the level of their enzyme activity. 

Consuming pro- and prebiotic products may further allow for the replacement of endogenous intestinal bacteria, which may be hazardous for human health, with well-characterized probiotic bacteria. Modulation of bacterial enzyme activity has been described as one of the mechanisms through which pre- and probiotics exert their beneficial effects [16]. 

*Bifidobacteria* and *Lactobacilli* are the most widely studied probiotic genera and have been shown to exert cancer protective effects in vitro and in vivo [17,18,19]. Several studies have also described a significant reduction in bacterial enzyme activity after probiotic administration [19,20]. Similarly, prebiotics have been shown to increase intestinal *Bifidobacteria* concentrations and to suppress fecal activities of carcinogen-metabolizing enzymes in humans [3,4]. 

While there are several carbohydrates accepted as prebiotics, there is still a need for new compounds with similar properties and possibly an easy production process. Nevertheless, the main goal of the prebiotic substance is to selectively, and to a considerable degree, promote the growth of the probiotic bacteria (e.g., *Lactobacillus* and *Bifidobacteria*), while not undergoing digestion by gastrointestinal enzymes [21,22]. 

In the food industry, the process of finding new functional foods or prebiotic candidates is certainly of great priority. Resistant dextrin was already successfully implemented in products, such as Fibelsol-2 or Nutriose, which are known for their beneficial effects and verified by several clinical trials [23]. In this study, a prebiotic candidate substance, namely resistant dextrin from potato starch, was evaluated. We tested whether the resistant dextrin can be utilized by the bacteria as a main source of carbon. 

Moreover, its influence on growth of selected intestinal bacteria was investigated to verify if it can selectively stimulate the growth of beneficial strains of gut bacteria, while negatively impacting others. Furthermore, activities of fecal enzymes (α-glucosidase, α-galactosidase, β-glucosidase, β-galactosidase and β-glucuronidase) were determined to investigate, whether the resistant dextrin can positively influence the host by decreasing activities of adverse enzymes (mainly β-glucosidase and β-glucuronidase), while not or only slightly affecting others.

## 2. Materials and Methods

### 2.1. Bacterial Isolates

The strains of intestinal bacteria *Lactobacillus*, *Bifidobacterium*, *Clostridium*, *Escherichia coli*, *Enterococcus* and *Bacteroides* were collected from the fecal samples taken from healthy children (both genders) aged 3–17 years (Table 1). Patients were not subjected to antibiotic therapy for at least 3 months prior to the collection of samples, had not received medications or supplements influencing the intestinal microbiome, nor had they reported episodes of diarrhea.

The inoculum of individual bacteria was prepared so that, after 24 h of cultivation, the number of cells of each species was in the range of 3.2 × 10^7^–4.5 × 10^7^ CFU/mL. Monocultures of *Lactobacillus* (MRS, Merck) *and Bifidobacterium* (MRS, Merck), *Clostridium* (VL, Merck) and *Bacteroides* (VL, Merck), *Escherichia coli* (nutritional broth) and *Enterococcus* (nutritional liquid broth) were cultivated. Strains of *Lactobacillus*, *Escherichia coli* and *Enterococcus* were incubated without oxygen restriction, whereas *Bifidobacterium*, *Bacteroides* and *Clostridium* were cultured under anaerobic conditions. 

After the incubation, the cultures were centrifuged (MPW-350R) at 9000 rpm for 10 min at 22 °C. The supernatant was removed, while the biomass from all cultures was transferred to modified with the addition of resistant dextrin medium of Wynne et al. (2004) [24] containing bile salts, 0.5 g/L; NaCl, 0.1 g/L; K_2_HPO_4_, 0.04 g/L; KH_2_PO_4_, 0.04 g/L; L-cysteine, 0.5 g/L; MgSO_4_x7H_2_O, 0.01 g/L; NaHCO3, 0.39 g/L; Tween, 2 g/L; peptone K, 10 g/L; MnSO_4_x4H_2_O, 0.01 g/L; hemin, 0.05 g/L; Vitamin K, 0.01 g/L; and resistant dextrin, 10 g/L. Control samples contained glucose at 10 g/100 mL instead of resistant dextrin. 

### 2.2. Growth Dynamics of the Intestinal Microbiota

The co-cultures were incubated for 168 h in conditions resembling those in the intestinal tract (anaerobic, 37 °C) in a Concept 400 Anaerobic Workstation (Baker Ruskinn, Sanford, ME, USA). The cultures were collected directly after the inoculation (0 h), then after 24, 48, 72, 96 and 168 h of incubation and diluted in physiological salt prior to selective plating (Koch’s plate method): *Lactobacillus* on Rogosa agar (Biomaxima, Lublin, Poland), *Bifidobacterium* on RCA agar (BTL, Lodz, Poland) with the addition of dicloxacillin (Sigma-Aldrich, Burlington, MA, USA), *Escherichia coli* on ENDO agar (Biomaxima, Poland), *Enterococcus* on bileaesculin agar (Biomaxima, Poland), *Clostridium* on DRCM agar (Biomaxima, Poland) and *Bacteroides* on Schaedler agar (Biomaxima, Poland) with Schaedler supplement (Biomaxima, Poland). 

Afterwards, the plates were incubated for 48 h at 37 °C; *Lactobacillus*, *Escherichia coli* and *Enterococcus* under aerobic conditions and *Bifidobacterium*, *Bacteroides* and *Clostridium* under anaerobic conditions in a Concept 400 Anaerobic Workstation (Ruskinn Biotrace, Baker Ruskinn, Sanford, ME, USA). All cultures were done in duplicates. Simultaneously, the changes in pH were monitored using an Elmetron CP-401 pH-meter (Elmetron, Zabrze, Poland).

### 2.3. Determination of Prebiotic Index

In order to assess the ability of *Bifidobacterium* and *Lactobacillus* bacteria to dominate in co-culture with *Clostridium* and *Bacteroides* in a medium supplemented with resistant dextrins, the prebiotic index (PI) was calculated. The equation (described below) considers changes in the key bacteria populations during fermentation and allows determination of the PI in vitro [25]. It is assumed that the increased counts of *Bifidobacteria* and *Lactobacilli* have a positive effect, as opposed to *Bacteroides* and *Clostridium*.

PI = (*Bifidobacterium*/Total bacteria) − (*Bacteroides*/Total bacteria) + (*Lactobacillus*/Total bacteria) − (*Clostridium*/Total bacteria),

where the unit of bacterial counts is log CFU/mL.

### 2.4. Enzymatic Assays

The activity of fecal enzymes: α-glucosidase, α-galactosidase, β-glucosidase, β-galactosidase and β-glucuronidase was determined by spectrophotometric methods. The methods used in the study were based on the reaction of 4-nitrophenyl α-D-glucopyranoside (Sigma-Aldrich, Burlington, MA, USA), 4-nitrophenyl β-d-glucopyranoside (Sigma-Aldrich, Burlington, MA, USA), 4-nitrophenyl α-d-galactopyranoside (Sigma-Aldrich, Burlington, MA, USA), 4-nitrophenyl β-d-galactopyranoside (Sigma-Aldrich, Burlington, MA, USA) and 4-nitrophenyl β-d-glucuronide (Sigma-Aldrich, Burlington, MA, USA) with α-glucosidase, β-glucosidase, α-galactosidase, β-galactosidase and β-glucuronidase, respectively. 

Each substrate was specific for the respective enzyme present in the sample. The reaction mixture contained 0.5 mL of phosphate buffer (pH = 7, 0.02 M), 0.05 mL of substrate solution (20 mM) and 0.25 mL of sample (post cultivation liquid). Incubation was performed at 37 °C, for 15 min (α-glucosidase, α-galactosidase and β-glucuronidase) or 60 min (β-glucosidase and β-galactosidase). 

Confirmation of the reaction was a change of sample color to yellow, while its intensity was directly proportional to the amount of released p-nitrophenol. The reactions were stopped using 0.25 M sodium carbonate. The absorbance was measured on the spectrophotometer Rayleigh UV-2601 (BFRL, Beijing, China) at a wavelength of λ = 400 nm. The unit of enzyme activity corresponds to the amount of p-nitrophenol (expressed in mM), which is released during 1 h of reaction, for 1 mg of protein in 1 mL of sample [µMh/g].

### 2.5. Statistical Analysis

The normality of the distribution of variables was examined with the Shapiro–Wilk test, and the homogeneity of variances was tested with Bartlett’s test. Following the confirmation of normality and equal variance, the results were analyzed using analysis of variance with a one-way ANOVA test and Tukey’s post hoc test. Statistical analysis was performed using Python at the significance level of *p* < 0.05. The results are presented as the mean ± standard deviation (SD).

## 3. Results

### 3.1. Growth Dynamics of the Gut Microbiota

The enzyme-resistant dextrin was tested as a source of carbon for probiotic *Lactobacilli* and *Bifidobacteria* cultured with other intestinal bacteria (*Clostridium*, *Escherichia coli*, *Enterococcus* and *Bacteroides*) isolated from the fecal samples of children (both genders) aged 3–17 years.

In a medium enriched with the resistant dextrin all the strains reached the stationary phase after the 24 h of cultivation. In the case of the genera *Lactobacillus* and *Bifidobacterium,* the counts of bacteria were similar and equal to 9.31 ± 0.77 and 9.33 ± 0.53 log CFU/mL, respectively (Table 2). After 168 h of incubation, the number of both *Lactobacillus* and *Bifidobacterium* remained high and amounted to 7.71 ± 0.81 and 7.79 ± 0.77 log CFU/mL, respectively. 

**Figure 1:**
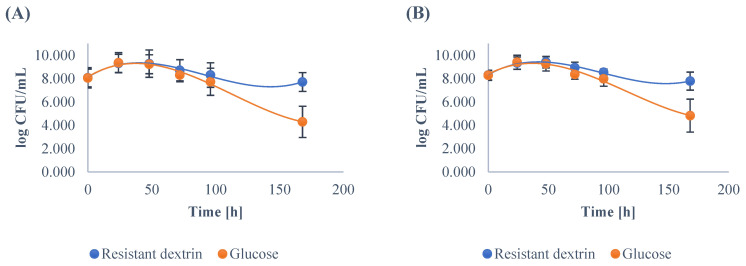
The growth of *Lactobacillus* (**A**) and *Bifidobacterium* (**B**) in the medium enriched with the resistant dextrin and with glucose as the control. The results show the means with standard deviations.

The minimum and maximum values of the number of *Lactobacillus* and *Bifidobacterium* after 168 h of cultivation were also relatively high and equal to 5.45 and 6.0 log CFU/mL (minimum); 8.73 and 8.85 log CFU/mL (maximum). These results suggest that the positive bacterial species were able to utilize the resistant dextrin for their growth and presented high viability in the co-culture with other strains (Figure 1). 

The control strains were cultivated on the medium with glucose instead of resistant dextrin. Similarly to the co-culture with resistant dextrin, the control strains reached the stationary phase after the 24 h of cultivation. The number of bacteria from the genera *Lactobacillus* and *Bifidobacterium* were similar to each other, as well as to the number of bacteria grown on the medium with resistant dextrin (Table 2). They amounted to 9.36 ± 0.86 and 9.41 ± 0.60 log CFU/mL, respectively. 

After 168 h of incubation, the number of both *Lactobacillus* and *Bifidobacterium* dropped significantly and was equal to 4.30 ± 1.34 and 4.83 ± 1.41 log CFU/mL, respectively. The minimum and maximum values of the number of *Lactobacillus* and *Bifidobacterium* after 168 h of cultivation were also noticeably lower and equal to 2.0 and 2.30 log CFU/mL (minimum); and 7.0 and 8.02 log CFU/mL (maximum). From these results, it may be concluded that cultivation on medium with glucose as a source of carbon causes a significantly faster decrease in the bacterial population (Figure 1). 

**Figure 2:**
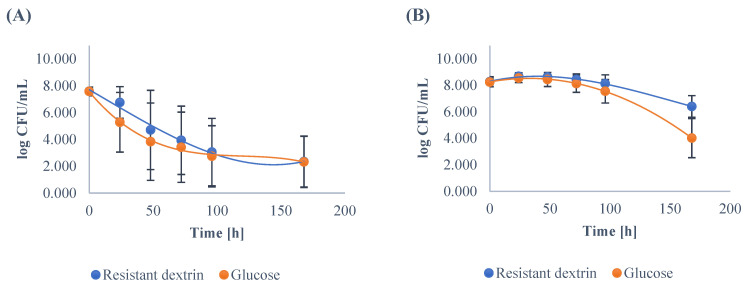
The growth of *E. coli* (**A**) and *Enterococcus* (**B**) in the medium enriched with the resistant dextrin and with glucose as the control. The results show the means and standard deviations.

Other genera of the intestinal bacteria were also able to grow successfully on the medium containing resistant dextrin; however, their ability to utilize the dextrin as a carbon source was more strain dependent. Successful growth was observed in the case of *Enterococcus* strains (Figure 2), where the number of cells in the stationary phase reached 8.64 ± 0.34 log CFU/mL. In the case of *E.coli,* the growth was altered in both media used. The growth phases were not well visible; therefore, no clear conclusion can be drawn from this data. 

**Figure 3:**
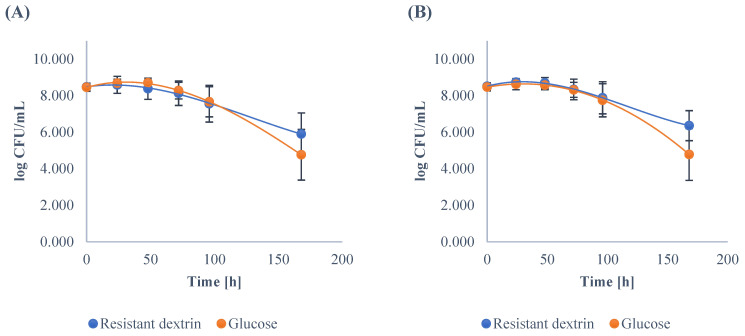
The growth of *Clostridium* (**A**) and *Bacteroides* (**B**) in the medium enriched with the resistant dextrin and with glucose as the control. The results show the means and standard deviations.

Strains of *Clostridium* and *Bacteroides* were similarly able to utilize resistant dextrin for growth and reached 8.38 ± 0.58 and 8.70 ± 0.30 log CFU/mL in their respective stationary phases (Figure 3). Moreover, the trend during their respective growth was similar to the *Enterococcus* genera. The counts of *Clostridium* and *Bacteroides* were significantly higher than in the case of control group (after 96 h of cultivation); however, it was still a less significant increase than the one measured for the *Lactobacillus* and *Bifidobacteria.*

The results indicated that the bacteria isolated from the feces of children were able to use the resistant dextrin as a source of carbon. The highest growth and the highest positive effect (increase in the number of bacteria over 168 h of culture) was noted for the genera *Lactobacillus* and *Bifidobacterium*. On the other hand, the weakest growth was reported for *Enterococcus* and *Escherichia coli*. We furthermore discovered that the stationary phase of *Lactobacillus* and *Bifidobacterium* strains lasted much longer than in the case of other intestinal bacteria. When the time of cultivation was prolonged to 168 h, there was a significant difference between the viability of the intestinal bacteria depending on the culture medium. 

In the case of medium enriched with resistant dextrin, the numbers of *Lactobacillus* and *Bifidobacterium* were 3.40 and 2.95 log cycles higher, respectively (Table 2). Unfortunately, the number of other bacteria also increased; however, it was much lower than in the case of probiotic strains: for *E. coli* 0.03 log cycle, for *Enterococcus* 2.4 log cycles, for *Clostridium* 1.1 log cycle and for *Bacteroides* 1.6 log cycle (Table 2). The total number of bacteria that grew on the medium with resistant dextrin after 168 h of cultivation was 11.5 log cycles higher than corresponding bacteria isolated from fecal samples that were grown on medium supplemented with glucose (Table 2). 

**Figure 4:**
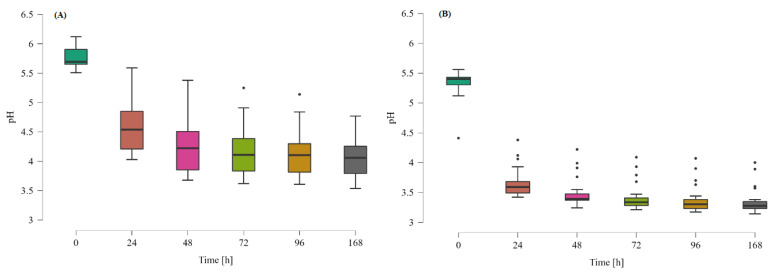
Boxplots presenting changes in the pH in the co-cultures grown on the medium enriched with the resistant dextrin (**A**) and with glucose as the control (**B**). The mean results from three repetitions for 30 samples. All results (**A**) are significantly different from results (**B**), which was tested by one-way ANOVA with post-hoc Tukey’s test (*p* < 0.05). Dots represent the outlier values.

In the medium supplemented with resistant dextrin, the pH was higher from the beginning of the cultivation; however, the decrease of pH during the logarithmic phase was noticeably milder than in the case of control with glucose (Figure 4). In the control samples, the pH dropped from 5.34 to 3.64 after the first 24 h, and then it was slowly decreasing through the cultivation time, down to 3.33 at 168 h of incubation. On the other hand, the decrease of pH on medium with resistant dextrin was lower and steadier. It dropped from 5.77 to 4.55 after the 24 h of cultivation and continued to decrease, until it reached its lowest value of 4.06 at 168 h of incubation. 

The substantial decrease in the pH in the case of culture with glucose as the source of carbon, could have been caused by the fact that glucose is a fundamental source of carbon for the cultured bacteria; therefore, the production of acidic metabolites (e.g., SCFA) was higher than in the case of bacteria grown on medium with resistant dextrin. Moreover, as glucose is a non-selective nutrient, it causes simultaneous growth of all kinds of bacteria, including the strongly acidifying *Bifidobacterium* and *Enterococcus* strains. 

Table 2, for the media with resistant dextrin, had positive values but were relatively low at the beginning of cultivation; however, its value grew with the time of culture (from 0.058 at 24 h of incubation to 0.163 at 168 h), which is a clear confirmation that beneficial bacteria from the genera *Bifidobacterium* and *Lactobacillus* can dominate the environment with other species of intestinal bacteria in the presence of resistant dextrin. The calculations of PI were done according to the results present in Table 2.

The significantly higher number of the probiotic bacteria in the co-culture could be caused by the multidimensional interactions between the microorganisms, where the neighboring strains exchange gases, nutrients and other compounds, which may further influence the growth dynamics in the culture. Fermentation of the resistant dextrin could also result in the formation of the hydrogen, which would be utilized and shared between coexisting bacterial species [26].

### 3.2. The Effect of Resistant Dextrin on the Activity of Fecal Enzymes

The analysis aimed to determine the influence of the resistant dextrin on the activity of fecal enzymes in the samples containing post cultivation liquid (described in the previous section). The main hypothesis was that the cultivation of the intestinal bacteria on the medium with resistant dextrin would cause a mild change in the activities of positive/or neutral fecal enzymes (e.g., α-glucosidase, α-galactosidase), while the activities of enzymes with a negative impact on health (β-glucosidase, β-galactosidase and β-glucuronidase) would be significantly decreased. 

Throughout the samples, regardless of the medium used for the cultivation, the activity of fecal enzymes increases with time. Nevertheless, the visible trend was that in the case of medium supplemented with resistant dextrin, the enzymatic activities were lower after the first 24 h of cultivation, and even though they increased with time, their final activity was lower than in the control samples (Table 3).

We found that the activity of α-glucosidase was noticeably lower in the liquid from the culture with resistant dextrin just after the 24 h and equal to 3.31 ± 1.19 µMh/g, whereas the activity in the control samples amounted to 4.33 ± 0.88 µMh/g. This trend continued throughout the samples; however, after the 168 h of cultivation, the difference between the test and control samples was significantly smaller (12.2% compared to 30.8% after the first 24 h) with the α-glucosidase activity of 4.71 ± 1.07 and 5.36 ± 0.88 µMh/g, respectively (Table 3). The highest activity of the enzyme was found in the samples from 168th h of cultivation and was equal in both test and control samples.

In the case of β-glucosidase, the highest activity of the enzyme was reported after the 96th h of cultivation, which amounted to 0.55 ± 0.21 µMh/g for the samples with resistant dextrin and 0.8 ± 0.26 µMh/g for the control. As opposed to the α-glucosidase, the activity of β-glucosidase differed considerably after the first 24 h of cultivation and after 168 h with the enzymatic activities being lower in the samples with resistant dextrin by 40% and 31%, respectively. This result suggests that the enrichment of medium with resistant dextrin contributed to statistically significant (*p* < 0.05) decrease in the activity of β-glucosidase. 

The activity of α-galactosidase was affected in the similar manner to the previous enzymes. The highest activity of the enzyme was achieved after the 96th h of cultivation and was equal to 3.87 ± 0.63 µMh/g for the samples with resistant dextrin and 4.76 ± 0.73 µMh/g for the control. After 24 h of incubation, the activity was on average 0.905 µMh/g lower (compared to control) in the samples with resistant dextrin and equal to 3.44 ± 0.70 µMh/g. Similarly, the final activity of α-galactosidase after 168 h of cultivation was, on average, 0.86 µMh/g lower for the samples with resistant dextrin and amounted to 3.66 ± 0.65 µMh/g (Table 3). 

In the case of β-galactosidase, the determined enzymatic activities were similar to those of β-glucosidase. After the first 24 h of incubation, the enzyme’s activity in the control group was higher and equal to 0.92 ± 0.19 µMh/g, whereas in the case of samples with resistant dextrin, the activity was 21.5% lower and equal to 0.73 ± 0.14 µMh/g. After 168 h of cultivation, the trend continued and the activities amounted to 0.97 ± 0.19 and 0.76 ± 0.13 µMh/g, for the control and test samples, respectively. The activity of β-galactosidase showed the lowest increase during the cultivation time; however, the final concentrations differed significantly, which again shows the influence of the resistant dextrin (Table 3). 

Evaluation of the activity of β-glucuronidase showed that, after the 24 h of cultivation, there was a significant difference between samples with the resistant dextrin compared to control. The activities were equal 2.55 ± 0.93 and 4.02 ± 0.83 µMh/g, respectively, which corresponds to a 36.7% difference. After 168 h of cultivation, the activity of β-glucuronidase was considerably lower in the case of samples with resistant dextrin and equal 3.20 ± 0.94 µMh/g, whereas for the control, the enzymatic activity was 35% higher and equal to 4.89 ± 1.01 µMh/g (Table 3). 

**Figure 5:**
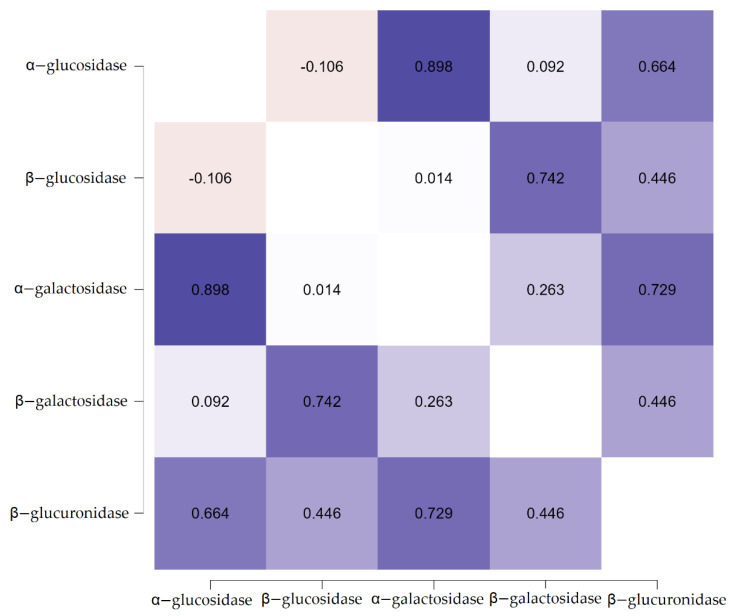
Graphical representation of correlations between the different fecal enzymes with a partial Pearson’s r heatmap.

The correlations between the activities of fecal enzymes were also analyzed in order to obtain further insights and possibly discover some trends (Figure 5). Predictably, there are strong correlations between the enzymatic activities of α-glucosidase and α-galactosidase and between β-glucosidase and β-galactosidase. The stronger the domination of the probiotic bacteria (*Lactobacillus* and *Bifidobacteria*), the higher the activities of enzymes produced by them. 

Surprisingly, there also is a significant positive correlation between α-glucosidase (and α-galactosidase) and β-glucuronidase. The majority of the obtained results can be explained by the fact that, due to the supplementation with resistant dextrin, the balance of intestinal bacteria shifts, and the growth of probiotic strains is promoted. The pH in the culture changes, which may also influence the activity of certain enzymes. It is also possible that the fermentation of the resistant dextrin produces a by-product that reacts and/or lowers the activity of fecal enzymes, such as β-glucosidase and β-glucuronidase.

## 4. Discussion

In the presented study, a dextrin resistant to the digestion by enzymes was used. This dextrin was produced under rigorously controlled conditions by the dextrinization of potato starch, which was acidified beforehand with hydrochloric and citric acid. The results obtained in this study are consistent with other similar studies [27,28,29]. 

The total number of bacteria grown on the medium with resistant dextrin after cultivation was significantly higher than the corresponding bacteria cultured on medium with the addition of glucose. Moreover, the growth of probiotic strains was notably more stimulated than the growth of other bacteria. Such an effect may have been caused by the protective properties of the resistant dextrin on the intestinal bacteria, which allowed for the higher viability of strains after time (72–168 h), which normally corresponds to the pathological passage of digested food [29]. 

Prebiotic properties of this kind of resistant dextrin were likewise described in earlier studies [26,30,31]. Similarly to the presented study, the investigated resistant dextrin was a viable source of carbon for the probiotic genera of intestinal bacteria, such as *Lactobacillus* and *Bifidobacterium*. We further reported that the growth of the *Clostridium* and *Bacteroidetes* was limited. Similar findings were also described quite recently in the study, where lactose-free milk was enriched with the resistant dextrin [27]. 

The study showed a significantly higher (35%) number of lactic acid bacteria (*Lactobacillus* and *Bifidobacterium*) compared with the numbers of strains from *Clostridium* and *Bacteroides* genera. The tests were done *in vitro*; however, the intestinal bacteria were also isolated from the fecal samples from human subjects of various ages. Furthermore, it demonstrated that the resistant dextrin is suitable as a food additive, which makes it viable for usage in the food industry for different kinds of health-promoting beverages or foodstuffs. 

Different sources of starch for the preparation of resistant dextrin are also viable. Studies show that resistant dextrin obtained from wheat or maize starch also presents prebiotic properties. A randomized, placebo-controlled trial on healthy patients conducted by Lefranc-Millot et al., (2012) [32] showed that NUTRIOSE (sugar-free resistant dextrin from what or maize starch) noticeably increased the numbers of beneficial bacteria (*Lactobacillus* and *Bifidobacterium*), whereas the counts of *Clostridium perfringens* were significantly reduced. Moreover, the impact on the fecal enzymes was also demonstrated with increased activity of β-glucosidase after the consumption of NUTRIOSE. 

Complementary results were achieved in the case of other resistant dextrin available on the market, Fibersol-2. A study of Burns et al., (2018) [33] on the effects of the resistant maltodextrin on growth of beneficial bacteria demonstrated that it stimulates the growth of *Bifidobacteria* after only 3 weeks. The dose required for the effects was 15 g of dextrin/day, which is even less than the recommended fiber intake for an adult [34]. 

Another recent study conducted by Hess et al., (2020) [35], which investigated the influence of dietary fiber (Fibersol-2) with a low energy diet on body weight and composition of the intestinal microbiota found that the resistant maltodextrin positively affected the number of *Parabacteroides* and *Bifidobacteria.* During this 12-week long intervention, overweight patients received supplementation with a total of 20 g of fiber/day. Although a significant weight loss was not reported, beneficial changes in the gut microbiota transpired, which further demonstrates the efficiency of resistant dextrins.

Similar findings were demonstrated in the in vitro study of Barczynska et al., (2016) [36], which aimed to assess if the resistant dextrin from maize starch is able to stimulate the growth of *Bacteroides* and *Actinobacteria* (representing the dominant microbiota of lean individuals) and simultaneously decrease the number of *Firmicutes* associated with obese subjects. The dextrins obtained from maize starch were able to stimulate the growth of beneficial bacterial strains and, moreover, were able to increase the concentration of SCFA, which gives them health-promoting value.

Another in vitro study demonstrated the effects of wheat dextrin on the growth of beneficial strains of intestinal bacteria from *Lactobacillus* and *Bifidobacterium* genera [21]. The study results clearly indicated that the wheat dextrin promoted the growth of the probiotic strains and increased the concentration of SCFAs in the first 24 h of cultivation. 

As mentioned before, fecal enzymes (e.g., β-glucosidase and β-glucuronidase) can be potentially harmful for the host organism and can promote the formation of several types of cancer [20]. Therefore, it is a matter of high importance to control their activities. Supplementation with probiotics and prebiotics appears to be a valid option in successfully performing this task as changing the balance of the intestinal microbiota affects the activities of fecal enzymes available for the digestion of carbohydrates [9,14,37]. 

In this study, a beneficial alteration in the enzymatic activities of fecal enzymes was recorded. The obtained results indicate that a resistant dextrin had a significant beneficial effect on the modulation of the activity of fecal enzymes produced by intestinal microbiota obtained from the fecal samples of children. This statement was especially true for the β-glucosidase and β-glucuronidase, as these had their activities significantly reduced in the samples cultured with the addition of resistant dextrin as compared to the control samples cultivated with glucose. 

Such influence of the resistant dextrin is considered to be beneficial for the host, as β-glucosidase (similarly to β-glucuronidase) is known for the conversion of heterocyclic aromatic amines, polycyclic aromatic hydrocarbons and some bile acids to carcinogenic compounds and production of for example aglycons [20,38,39]. Moreover, the inhibition of β-glucosidase was shown to increase the sensitivity of colorectal cancer to chemotherapy and suppress the growth of cancer [40].

The results concerning the activity of β-glucuronidase were a promising discovery as well, due to the association of this enzyme with the formation of colorectal cancer [41,42]. Other enzymes were also affected; however, to a lesser extent, which indicates the potential of resistant dextrin from potato starch to reduce the risk of developing colorectal cancer. 

The number of studies that directly investigated the effects of resistant dextrin on the activity of fecal enzymes is relatively low. Moreover, these studies often explore the properties of widely available preparations, such as NUTRIOSE, while omitting other sources of resistant dextrin [43,44]. Their results indicate that daily administration of resistant dextrins for 4–5 weeks increased the activity of α-glucosidase and β-glucosidase. 

Such an effect may be considered positive in the case of α-glucosidase, since it causes benefits ability to digest more fiber, thus, producing more SCFA. On the other hand, increased β-glucosidase activity can lead to formation of colon cancer, although it can also release several flavonoids with antioxidative and antimutagenic effects [44]. 

Nonetheless, the resistant dextrin used in the current study notably increases the viability of probiotic strains, such as *Lactobacillus*, which were reported to decrease the activity of fecal enzymes in several studies [45,46,47]. The results showed that probiotic *lactobacilli* (*L. casei*, *L. plantarum* and *L. rhamnosus)* were able to drastically reduce the activity of fecal β-glucuronidase in human subjects by up to 58%. Such findings indicated significant anti-carcinogenic effects [45]. 

Similarly, the study conducted by [46] proved that the combination of *L. plantarum* and prebiotic (acacia gum) significantly reduced the activities of procarcinogenic β-glucosidase and β-glucuronidase even more than supplementation with either probiotic or prebiotic alone. Other positive effects observed in the study concerned reinforced levels of immunoglobulins, reduced cholesterol and increased antioxidant activity in the liver.

Furthermore, there are studies that indirectly confirm the effects of probiotic strains of *Bifibacteria* and *Lactobacillus* genera [47,48]. In these separate studies, it was reported that strains of *L. brevis* (isolated from kimchi), *B. bifidum* and *B. longum* (isolated from feces of infants) did not possess β-glucuronidase activity. Such findings suggest that the increased abundance of this genera of bacteria in the gastrointestinal tract may be followed by decreased levels of the procarcinogenic glucuronidases. 

Probiotic strains of *Bifidobacterium* were furthermore able to decrease the activity of harmful fecal enzymes. A study of Kim et al., (2008) [49] showed that *Bifidobacterium adolescentis* isolated from fecal samples from healthy Korean subjects inhibited the activity of fecal urease, glucuronidase and glucosidase, thus, having anti-carcinogenic properties. 

The results of this study indicate that the resistant dextrin from potato starch is a viable source of carbon for fecal bacteria. Moreover, it promotes the growth of the beneficial *Lactobacillus* and *Bifidobacterium* strains to a much higher degree than in the case of other intestinal bacterial species, which is clearly demonstrated by the positive value of the prebiotic index (PI) at the end of cultivation. 

Simultaneously, it lowers the activity of fecal enzymes (α-glucosidase, α-galactosidase, β-glucosidase, β-galactosidase and β-glucuronidase); however, the higher inhibition was reported in the case of the potentially harmful and pro-carcinogenic β-glucuronidase and β-glucosidase. The effect of the resistant dextrin on the pH was not significant; however, the noticeable minor effects should not be disregarded as they could potentially play a role in establishing a beneficial balance of intestinal microbiota. 

This study was focused on the genera of bacteria isolated from the fecal samples of children; however, we did not focus on the specific species of bacteria. Future studies engaging in the topic of prebiotic properties of the resistant dextrin from potato starch could potentially evaluate its properties on specific probiotic strains of *Lactobacillus* and *Bifidobacterium* in an environment with other bacteria commonly found in the large intestine. Such studies would give better insight into the matter of selectivity of the resistant dextrin and could further specify if it promotes the growth of every probiotic *Lactobacillus* and *Bifidobacterium* strain or is biased in this regard. 

## 5. Conclusions

In conclusion, the effects of the investigated resistant dextrin on the growth dynamics and the activity of fecal enzymes are considerable and can be perceived as beneficial. The growth of intestinal bacteria isolated from children’s feces was possible on medium enriched with the resistant dextrin, which indicates that it can be used as a main source of carbon for these bacteria. Moreover, the results proved that, in the presence of resistant dextrin, the balance of the probiotic strains changes, and the viability of the beneficial strains is maintained longer. 

Furthermore, the post-cultivation liquid from samples cultivated with the addition of resistant dextrin to the culture medium contained enzymes of lesser activity, where the most significant decrease in the enzymatic activity was reported for the β-glucosidase and β-glucuronidase. The activities of other enzymes were also affected; however, their drop was not as considerable, and their overall activity remained relatively high. Therefore, based on the obtained results, it can be stated that the elaborated effect of resistant dextrin is potentially beneficial for the host. Nevertheless, in vivo studies are required to obtain confirmation of these positive effects.

## Figures and Tables

**Table 1:** Summary of information about the origins of the bacterial isolates.

Age [Years]	Participants		BMI ≤ 25		BMI ≥ 25	
	Male	Female	Male	Female	Male	Female
3–7	7	6	100%	100%	0%	0%
8–12	5	6	60%	83%	40%	17%
13–17	5	8	40%	12%	60%	88%
Total	17	20	29%	60%	71%	40%

Percentages shown in the table refer to the respective gender groups.

**Table 2:** Comparison of growth of the intestinal bacteria in the samples cultivated on medium with the resistant dextrin and glucose (control) as a source of carbon.

	Resistant Dextrin						Glucose					
	*Lactobacillus*											
Time [h]	0	24	48	72	96	168	0	24	48	72	96	168
Minimum	5.22	6.00	3.56	4.56	3.08	5.48	5.61	6.00	6.00	6.00	2.30	2.00
Maximum	8.79	9.94	9.99	9.83	8.96	8.73	8.92	9.96	9.99	8.84	8.68	7.00
Mean	8.07	9.30	9.29	8.72 ^A^	8.31 ^A^	7.71 ^A^	8.06	9.36	9.24	8.31 ^B^	7.73 ^B^	4.29 ^B^
SD	0.87	0.77	1.17	0.91	1.05	0.81	0.76	0.86	0.81	0.57	1.16	1.34
	*Bifidobacterium*											
Minimum	7.15	8.26	8.36	7.92	7.85	6.00	7.06	8.24	7.60	7.34	6.00	2.30
Maximum	8.69	9.92	9.99	9.91	9.06	8.85	8.73	9.99	9.96	8.90	8.65	8.02
Mean	8.30	9.33	9.42 ^A^	8.94 ^A^	8.55 ^A^	7.79 ^A^	8.28	9.41	9.26 ^B^	8.36 ^B^	7.98 ^B^	4.83 ^B^
SD	0.42	0.53	0.49	0.47	0.28	0.77	0.43	0.60	0.61	0.41	0.63	1.41
	*E. coli*											
Minimum	6.67	4.18	0.00	0.00	0.00	0.00	6.60	0.00	0.00	0.00	0.00	0.00
Maximum	8.09	8.34	8.00	7.50	7.13	5.62	8.15	8.16	7.28	6.83	5.90	5.37
Mean	7.59	6.76 ^A^	4.71 ^A^	3.94 ^A^	3.06 ^A^	2.35	7.59	5.28 ^B^	3.83 ^B^	3.42 ^B^	2.75 ^B^	2.32
SD	0.29	1.18	2.96	2.55	2.51	1.90	0.33	2.23	2.88	2.62	2.28	1.91
	*Enterococcus*											
Minimum	7.48	7.93	7.54	7.11	6.30	5.00	7.51	7.68	6.60	6.00	5.81	0.00
Maximum	8.88	9.02	9.00	8.85	8.79	8.03	8.79	8.91	8.89	8.81	8.51	6.69
Mean	8.28	8.68	8.64 ^A^	8.44 ^A^	8.13 ^A^	6.41 ^A^	8.24	8.55	8.44 ^B^	8.13 ^B^	7.56 ^B^	4.02 ^B^
SD	0.38	0.28	0.34	0.42	0.66	0.82	0.33	0.34	0.53	0.66	0.89	1.48
	*Clostridium*											
Minimum	7.93	7.05	6.77	6.18	5.53	4.22	8.05	8.29	8.27	6.89	5.66	2.00
Maximum	8.90	9.12	8.96	8.84	8.62	8.47	8.79	9.47	8.97	8.79	8.76	7.58
Mean	8.48	8.59	8.38 ^A^	8.13 ^A^	7.56	5.89 ^A^	8.46	8.68	8.70 ^B^	8.27 ^B^	7.66	4.76 ^B^
SD	0.21	0.47	0.58	0.67	1.00	1.15	0.22	0.22	0.16	0.45	0.82	1.39
	*Bacteroides*											
Minimum	8.02	8.16	7.84	7.00	5.49	5.11	8.10	7.87	7.94	7.45	5.53	2.00
Maximum	8.86	9.00	9.06	9.06	8.92	8.18	8.87	9.46	9.03	8.76	8.95	7.62
Mean	8.51	8.74 ^A^	8.69 ^A^	8.34	7.88 ^A^	6.36 ^A^	8.47	8.62 ^B^	8.57 ^B^	8.31	7.74 ^B^	4.78 ^B^
SD	0.19	0.20	0.30	0.56	0.88	0.82	0.21	0.30	0.25	0.41	0.91	1.42

The mean results from three repetitions for 30 samples. The results are significantly different from: ^A^ the control group; ^B^ the resistant dextrin group; one-way ANOVA with post-hoc Tukey’s test (*p* < 0.05). SD: Standard deviation; and Unit: log CFU/mL.

**Table 3:** Comparison of activities of the fecal enzymes in the samples cultivated on medium with the resistant dextrin or glucose (control) as a source of carbon.

	Resistant Dextrin					Glucose				
	α-glucosidase									
Time [h]	24	48	72	96	168	24	48	72	96	168
Minimum	0.51	1.11	1.76	2.54	2.72	2.77	3.09	3.44	3.86	3.95
Maximum	5.41	5.72	6.07	6.49	6.58	5.62	5.94	6.28	6.79	6.92
Mean	3.31 ^C^	3.69 ^C^	4.10 ^C^	4.60 ^C^	4.71 ^C^	4.33 ^D^	4.61 ^D^	4.95 ^D^	5.32 ^D^	5.36 ^D^
SD	1.19	1.15	1.11	1.08	1.07	0.88	0.85	0.84	0.86	0.88
	β-glucosidase									
Minimum	0.04	0.15	0.19	0.16	0.16	0.19	0.30	0.34	0.31	0.30
Maximum	0.76	0.87	0.91	0.88	0.87	1.14	1.17	1.19	1.28	1.24
Mean	0.39 ^C^	0.52 ^C^	0.57 ^C^	0.55 ^C^	0.55 ^C^	0.65 ^D^	0.75 ^D^	0.78 ^D^	0.80 ^D^	0.79 ^D^
SD	0.22	0.21	0.21	0.21	0.21	0.28	0.24	0.23	0.26	0.25
	α-galactosidase									
Minimum	1.35	1.56	1.75	2.25	2.00	3.16	3.22	3.25	3.47	3.25
Maximum	4.42	4.48	4.51	4.73	4.51	5.79	5.89	5.95	6.17	5.87
Mean	3.44 ^C^	3.53 ^C^	3.60 ^C^	3.87 ^C^	3.66 ^C^	4.34 ^D^	4.44 ^D^	4.50 ^D^	4.76 ^D^	4.52 ^D^
SD	0.70	0.68	0.66	0.63	0.65	0.73	0.73	0.74	0.73	0.72
	β-galactosidase									
Minimum	0.41	0.49	0.56	0.57	0.51	0.60	0.66	0.68	0.74	0.70
Maximum	0.94	1.00	1.00	1.01	1.00	1.26	1.28	1.30	1.37	1.32
Mean	0.73 ^C^	0.75 ^C^	0.77 ^C^	0.78 ^C^	0.76 ^C^	0.92 ^D^	0.95 ^D^	0.97 ^D^	0.99 ^D^	0.97 ^D^
SD	0.14	0.14	0.13	0.12	0.13	0.19	0.19	0.19	0.20	0.19
	β-glucuronidase									
Minimum	0.43	0.45	0.75	1.10	1.23	2.35	2.61	2.72	2.79	2.84
Maximum	4.19	4.59	4.82	5.04	5.13	5.73	5.87	6.68	6.71	6.94
Mean	2.55 ^C^	2.84 ^C^	2.99 ^C^	3.13 ^C^	3.20 ^C^	4.02 ^D^	4.27 ^D^	4.67 ^D^	4.76 ^D^	4.89 ^D^
SD	0.93	0.96	0.95	0.94	0.94	0.83	0.83	0.96	0.96	1.01

The mean results from three repetitions for 30 samples. The results are significantly different from: ^C^ the control group; ^D^ the resistant dextrin group; one-way ANOVA with post-hoc Tukey’s test (*p* < 0.05). SD: Standard deviation; and Unit: µMh/g.

## Data Availability

The data analyzed are publicly available in source articles and data citations were included in the reference list. The data that support the findings of this study is also available from the corresponding author, K.Ś. and M.W.
